# Modeling of atrophy size trajectories: variable transformation, prediction and age-of-onset estimation

**DOI:** 10.1186/s12874-021-01356-0

**Published:** 2021-08-17

**Authors:** Charlotte Behning, Monika Fleckenstein, Maximilian Pfau, Christine Adrion, Lukas Goerdt, Moritz Lindner, Steffen Schmitz-Valckenberg, Frank G Holz, Matthias Schmid

**Affiliations:** 1 Department of Medical Biometry, Informatics and Epidemiology, University Hospital Bonn, Venusberg-Campus 1, Bonn, 53127 Germany; 2 John A. Moran Eye Center, University of Utah, Salt Lake City, USA; 3 Ophthalmic Genetics and Visual Function Branch, National Eye Institute, Bethesda, MD USA; 4 Institute for Medical Information Processing, Biometry and Epidemiology, Ludwig-Maximilians-University, Munich, Germany; 5 Department of Ophthalmology, University Hospital Bonn, Bonn, Germany

**Keywords:** Geographic atrophy, Age-related macular degeneration, Box-Cox transformation, Mixed-effects models, Prediction, Age-of-onset estimation

## Abstract

**Background:**

To model the progression of geographic atrophy (GA) in patients with age-related macular degeneration (AMD) by building a suitable statistical regression model for GA size measurements obtained from fundus autofluorescence imaging.

**Methods:**

Based on theoretical considerations, we develop a linear mixed-effects model for GA size progression that incorporates covariable-dependent enlargement rates as well as correlations between longitudinally collected GA size measurements. To capture nonlinear progression in a flexible way, we systematically assess Box-Cox transformations with different transformation parameters *λ*. Model evaluation is performed on data collected for two longitudinal, prospective multi-center cohort studies on GA size progression.

**Results:**

A transformation parameter of *λ*=0.45 yielded the best model fit regarding the Akaike information criterion (AIC). When hypertension and hypercholesterolemia were included as risk factors in the model, they showed an association with progression of GA size. The mean estimated age-of-onset in this model was 67.21±6.49 years.

**Conclusions:**

We provide a comprehensive framework for modeling the course of uni- or bilateral GA size progression in longitudinal observational studies. Specifically, the model allows for age-of-onset estimation, identification of risk factors and prediction of future GA size. A square-root transformation of atrophy size is recommended before model fitting.

**Supplementary Information:**

The online version contains supplementary material available at (10.1186/s12874-021-01356-0).

## Background

Age-related macular degeneration (AMD) is a leading cause of blindness, especially for people in developed countries older than 60 years [1, 2]. AMD has two late stages: choroidal neovascularization (CNV) and geographic atrophy (GA). Here we consider GA, which is thought to be the end stage of AMD when CNV does not develop [3] and which is responsible for vision loss in approximately 20% of all patients with AMD [4]. More than five million people are estimated to be affected by GA worldwide, a number which is supposed to increase with the aging of the population [2]. To date, there is no effective standard treatment available [5].

GA is defined by atrophic lesions of the outer retina resulting from loss of retinal pigment epithelium (RPE), photoreceptors and underlying choriocapillaris (reviewed by [6]). These areas enlarge with time and lead to irreversible loss of visual function [7]. A relevant clinical measure of disease progression is the eye-specific size of GA which can be quantified based on imaging techniques including color fundus photography, spectral domain optical coherence tomography imaging, or fundus autofluorescence (FAF) imaging [8, 9].

A better understanding of the risk factors that accelerate GA size progression is necessary for the development of treatment options, in particular for the design of (interventional) clinical trials. To date, empirical evidence on GA size progression is usually collected through longitudinal observational studies (e.g. [10–12]). In these studies, it is essential to analyze GA size trajectories over time using an adequate statistical model. Specifically, in the absence of a randomized study design, data analysis needs to account for confounding issues as well as correlation patterns, for instance when both eyes of a patient are included in the study. In the latter case, the correlations between the eyes within one patient need to be incorporated as well as the correlations due to repeated measurements over time.

The aim of this analysis is to systematically derive a statistical approach for modeling GA size in observational ophthalmologic studies. As will be demonstrated in the following sections, the proposed approach generalizes various statistical models for GA size progression that have been used in previous publications (see below). Special focus will be given to the following issues, which are considered to be of particular importance for the planning and design of future interventional trials:

**(i) Transformation of GA size.** Before model fitting, it is important to consider whether the response (here, GA size) should be transformed. Finding an appropriate transformation can provide information about the underlying natural processes that drive the progression of GA. In recent publications on GA size progression, there has been an ongoing discussion about the optimal choice of transformation [11, 13–15]. Three main modeling paradigms have emerged: The first set of models assumes a linear relationship between GA size and covariables (e.g. risk factors or confounding variables). This implies a constant enlargement of GA size over time. Examples of this modeling approach can be found in [13, 14]. The second approach assumes a quadratic enlargement of the lesion size. This is motivated by the thought of circular atrophic lesions that constantly enlarge with their radiuses [11, 15]. The third model type is an exponential model in which atrophic lesions enlarge exponentially. Compared to a linear growth model, Dreyhaupt et al. [13] found that the assumption of exponential growth led to improved model fits.

**(ii) Age-of-onset estimation.** Another relevant topic for modeling GA size progression is the estimation of the age of disease onset. Research on this topic is motivated by the fact that in many clinical trials patients can only be included when the disease is already manifested in a later stage. The estimated age-of-onset may, in contrast to lesion size, be considered as time-invariant variable, and facilitate association analyses with other time-invariant variables such as the genotype.

**(iii) Identification of risk factors and confounding variables.** For the development of AMD treatments, it is essential to specify meaningful inclusion and exclusion criteria for use in future clinical trials. It is therefore of high importance to identify relevant risk factors and confounding variables, and to analyze their relationships with GA size progression. Such an analysis can be achieved by building a multivariable regression model from observational data that includes relevant risk factors and confounders as covariables.

To address the issues described above, we derive a statistical regression model that includes (possibly transformed versions of) GA size as response variable, as well as potential risk factors and/or confounders (such as e.g. age, smoking) as covariables. To account for the above mentioned correlations between eyes of the same patient as well as temporal correlations, we investigate the use of a mixed-effects modeling approach with patient- and eye-specific random effects terms. In this framework, we identify the “optimal” transformation of GA size by conducting a systematic search within the family of Box-Cox transformations [16]. As will be shown, this systematic approach also allows for the derivation of formulas for age-of-onset estimation. Furthermore, we demonstrate how predictions of future (untransformed) GA size values can be obtained from the fitted regression model.

For model derivation and illustration, we will apply the proposed methods to a data set collected by the multi-center *Fundus Autofluorescence in AMD* (FAM) study (NCT00393692) and by its single-center extension study, the *Directional Spread in Geographic Atrophy* (DSGA) study (NCT02051998). These noninterventional, prospective natural history studies adhered to the tenets of the Declaration of Helsinki and were approved by the institutional review boards of the participating centers. Written informed consent was obtained from each participant after explanation of the studies’ nature and possible consequences of participation.

## Methods

### Data

The data set used here was collected from patients with GA secondary to AMD that were recruited for the FAM study and followed-up in the DSGA study.

The inclusion and exclusion criteria have been described elsewhere [14, 17]. In brief, the two studies included eyes without any history of retinal surgery, radiation therapy, laser photocoagulation or retinal diseases other than AMD. GA size measurements were obtained by grading FAF retinal images that were recorded at the baseline and follow-up visits. Data was only used for statistical analysis if the difference in total GA size between two graders was smaller than 0.15mm^2^ and if the patients had at least two visits.

Our analysis data set contained *N*=150 eyes from *n*=101 patients that where examined in up to nine follow-up visits. At baseline, the median age was 75.7 years (IQR: 70.7−80.6 years); 61.4*%* of the patients were female, and the mean follow-up time was 3.36 years (range 0.5−13.7 years) due to the extension by the second study. The GA size varied strongly between eyes: mean GA size at baseline was 5.64mm^2^, ranging between 0.07mm^2^ and 31.41 mm^2^. The status of hypertension and hypercholesterolemia was assessed by a patient-reported questionnaire at the baseline visit. Information was obtained based on patients’ reports and current medication; medical reports were included in the assessment if available. For details see Table 1.

**Table 1:** Characteristics of the analysis data set used for statistical modeling

	Count	Percent
Patients (n)	101	
Eyes (N)	150	
Bilateral GA	49	48.50%
Unilateral GA	52	51.50%
Hypertension		
yes	56	55.40%
no	44	38.60%
Hypercholesterolemia		
yes	28	27.70%
no	70	69.30%
No. of patients with no. of visits		
2 visits	25	24.75%
3 visits	23	22.78%
4 visits	23	22.78%
5-9 visits	30	29.70%
	Mean (Range)	Median (IQR)
Age at baseline	75.61	75.66
[years]	(57.23 - 95.06)	(70.67 - 80.62)
Follow-up time	3.36	2.90
[years]	(0.50 - 13.70)	(1.61 - 4.57)
GA size at baseline	5.64	4.30
mm^2^	(0.07 - 31.40)	(1.76 - 7.60)

All data considered in this paper was collected from patients with GA secondary to AMD that were recruited for the FAM study. If further monitoring of these patients was performed via the DSGA study, the further progression is included in the analysis data set

### Regression modeling

Within a typical ophthalmologic study setting, patients participate in several follow-up visits at which one or both eyes are examined. This leads to correlated measurements, both within the patients and over time. Thus, a model is needed that captures complex correlation structures. A popular regression model, which has been used regularly in the literature on GA [11, 13] and which is also considered here, is a mixed-effects model with random effects terms for both eye and patient. Yet, there exists a variety of model specifications and the specific structure is still a matter of debate [18].

Before introducing the full mixed-effects model with possible risk factors and confounders, we start with a model that contains a time trend as only (continuous) covariable. This model serves as a basic model that captures the time dependency of GA enlargement.

**Mixed-effects model with time as only covariable.** As suggested by Shen et al. [18], we follow the hypothesis that the progression of GA has an underlying process of GA expansion that is mostly the same over time for all eyes. Differences in eyes may arise due to different exposition to environmental conditions, and, most importantly, GA size varies between patients as they enter the study at different time points in their disease history. We therefore propose to include the disease age *Δ*_*i*_≥0 of an eye *i* at study entry directly in the model. We further assume that the atrophy size *y*_*it*_ of an eye *i* depends on the (unknown) age of the disease at study entry *Δ*_*i*_ and the (observable) follow-up time *t*≥0 that has passed since. Time is assumed to be measured on a continuous scale, e.g. in days or years since baseline. Under the assumptions by Shen et al. [18], and considering (for the moment) a linear enlargement of GA, this leads to the following regression model:

1 $$ y_{{it}} = \beta \cdot (\Delta_{i} +t) + \epsilon_{{it}},   $$

where *β* denotes the regression slope (i.e. the constant enlargement rate). The residuals *ε*_*it*_,*i*=1,…,*N*, are assumed to be normally distributed with zero mean and variance *σ*^2^.

If it is further assumed that the disease age at study entry can be approximated by a normal distribution, the model in (1) can be parameterized such that it becomes a linear mixed-effects model. This is seen by defining $\theta _{i} := \beta \cdot \Delta _{i} \sim \mathcal {N}\left (\mu _{\theta }, \sigma ^{2}_{\theta }\right)$ and $ \alpha _{i} := \theta _{i} - \mu _{\theta } \sim \mathcal {N}\left (0, \sigma ^{2}_{\theta }\right)$, so that Model (1) can be written as

2 $$ y_{{it}} = \mu_{\theta} + \beta t + \alpha_{i} + \epsilon_{{it}}.   $$

In this form, the model reads as follows: The atrophy size *y*_*it*_ depends on a fixed intercept *μ*_*θ*_, an eye-specific random intercept *α*_*i*_ that reflects the deviation of the disease age of eye *i* at study entry from the mean disease age at study entry, and an overall linear time trend *βt* that is the same for all eyes.

When there are patients in the study that contributed data from both eyes, one needs to consider the nested data structure and account for the correlations between measurements taken from the same patient. This can be done by extending the model equation as follows:

3 $$ y_{{ijt}} = \mu_{\theta} + \beta t + \zeta_{j} + \alpha_{i} +\epsilon_{{ijt}},   $$

where $\zeta _{j} \sim \mathcal {N}\left (0, \sigma _{\zeta }^{2}\right), j=1,\ldots,n$, is a normally distributed patient effect and *α*_*i*_ the effect of an ’eye within a patient’. Note: While it is assumed that the residual terms *ε*_*ijt*_ are independent of the random effects *α*_*i*_ and *ζ*_*j*_, the latter two terms are generally allowed to be correlated. For simplicity, and without loss of generality, we will assume independence of all random effects terms in the following.

**Mixed-effects model with covariables.** When introducing covariables into the model, it is reasonable to assume that risk factors and/or confounders equally influence the enlargement of GA before and after inclusion of an eye in the study. This assumption can be incorporated in Model (1) by adding a covariable-dependent slope to the model equation:

4 $$ y_{{it}} = \left(\beta + \boldsymbol{\beta}_{x}^{\intercal} \boldsymbol{x}_{i}\right) \cdot (\Delta_{i} + t) + \epsilon_{{it}},  $$

where $\boldsymbol {x}_{i} = (x_{1},..., x_{k})^{\intercal }_{i}$ is a vector of *k* (possibly time-dependent) risk factors for each eye and $\boldsymbol {\beta }_{x} = \left (\beta _{x_{1}},..., \beta _{x_{k}}\right)^{\intercal }$ is a vector of parameters that accelerate or slow down GA size progression ($\beta _{x_{s}} > 0$ and $\beta _{x_{s}} < 0$, respectively, *s*∈{1,…*k*}). Note that in the following, we will not distinguish between risk factors and confounders any more, as we assume that both are collected in the vectors ***x***_*i*_.

Similar to the reparametrization used above, we write $ \Delta _{i} := (\mu _{\Delta } + \gamma _{i}) \sim \mathcal {N}\left (\mu _{\Delta }, \sigma _{\Delta }^{2}\right)$, where *μ*_*Δ*_ and $\sigma _{\Delta }^{2}$ denote the mean and the variance of the *i*-the eye at study entry.

The mixed-effects model with covariables can then be written as

5 $$ {}\begin{aligned} y_{{it}} &= \left(\beta + \boldsymbol{\beta}_{x}^{\intercal} \boldsymbol{x}_{i}\right) \mu_{\Delta} + \left(\beta + \boldsymbol{\beta}_{x}^{\intercal} \boldsymbol{x}_{i}\right) \gamma_{i} + \beta t + \boldsymbol{\beta}_{x}^{\intercal} \boldsymbol{x}_{i} t + \epsilon_{i t} \\ &= \beta \mu_{\Delta} + \beta t + \mu_{\Delta} \boldsymbol{\beta}_{x}^{\intercal} \boldsymbol{x}_{i} + \boldsymbol{\beta}_{x}^{\intercal} \boldsymbol{x}_{i} t + \beta \gamma_{i} + \boldsymbol{\beta}_{x}^{\intercal} \boldsymbol{x}_{i} \gamma_{i} + \epsilon_{i t}.  \end{aligned}  $$

with eye-specific random effects $\gamma _{i} \sim \mathcal {N}\left (0, \sigma _{\Delta }^{2}\right)$. The linear enlargement in Model (5) thus implies dependency of *y*_*it*_ on an interaction term between *t* and ***x***_*i*_, and also on random slopes of the covariable values ***x***_*i*_. Importantly, Eq. 5 implies numerous dependencies between the slope parameters associated with *t*, ***x***_*i*_,***x***_*i*_*t*,*γ*_*i*_, and ***x***_*i*_*γ*_*i*_, so that the model no longer possesses the structure of a “standard” mixed-effects model with unrestricted estimation of coefficients. Details on model fitting will be given below.

Finally, when considering patients that contribute data from both eyes, one specifies

6 $$ \begin{aligned} y_{{ijt}} &= \beta \mu_{\Delta} + \beta t + \mu_{\Delta} \boldsymbol{\beta}_{x}^{\intercal} \boldsymbol{x}_{i} + \boldsymbol{\beta}_{x}^{\intercal} \boldsymbol{x}_{i} t + \beta \gamma_{i} \\ & + \boldsymbol{\beta}_{x}^{\intercal} \boldsymbol{x}_{i} \gamma_{i} + \beta \zeta_{j} + \boldsymbol{\beta}_{x}^{\intercal} \boldsymbol{x}_{i} \zeta_{j} + \epsilon_{{ijt}} \end{aligned}   $$

with patient-specific random effects $\zeta _{j} \sim \mathcal {N}\left (0, \sigma _{\zeta }^{2}\right), j=1,\ldots, n$, and an additional interaction term between ***x***_*i*_ and *ζ*_*j*_.

The model equations presented so far ascribe a linear relationship between time, risk factors, and GA size. In the following section, possible transformations are examined, so that the modeling approach is extended to model nonlinear progressions.

### Transformation of the response

**Fig. 1:**
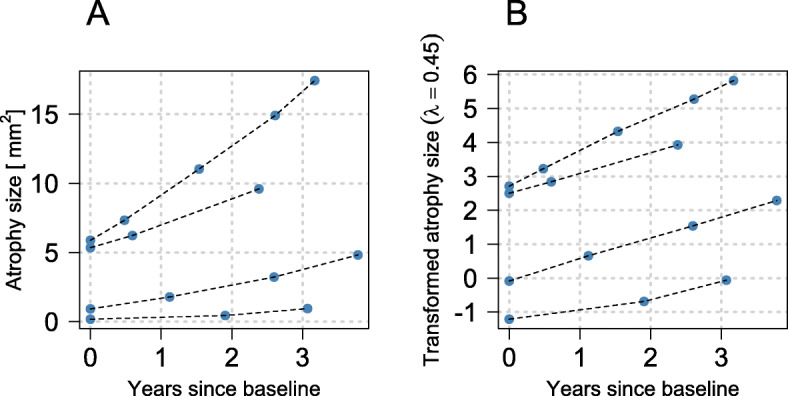
Progression of GA size. **A** Untransformed GA size trajectories of four different eyes contained in the analysis data set and **B** trajectories on a transformed scale with transformation parameter *λ*=0.45

As an example, Fig. 1 A shows the GA size trajectories of four eyes contained in the analysis data set. Considering these progressions, it is conceivable to assume that the trajectories are not strictly linear. Since the model equations above (Models (1) to (6)) refer to linear enlargement processes, a transformation of the response is convenient for modeling non-linear progression (see Fig. 1B).

Three different transformation approaches have been used in recent publications on GA size progression (e.g. [11, 13–15]): (i) Linear models with no response transformation implying a linear relationship between GA size and the covariables, (ii) linear models with square root transformation of the response, and (iii) linear models with log-transformed response – or equivalently exponentially transformed models with no transformed response – implying an exponential enlargement of the lesion size.

**Box-Cox transformation** Instead of comparing only the most commonly used transformations, we consider a systematic and more comprehensive strategy for finding an appropriate transformation of the GA size. For this systematic approach, the Box-Cox model class is applied because it covers a wide range of transformations, including the transformations (i) to (iii) above. More specifically, for an atrophy size *y*>0 we consider the class of Box-Cox transformations

7 $$ f_{\lambda}(y) := y^{(\lambda)} = \left\{\begin{array}{ll} \frac{y^{\lambda} -1}{\lambda} & \text{ if}\ \lambda \neq 0 \text{,} \\ \log(y) & \text{ if}\ \lambda = 0 \text{,} \end{array}\right.  $$

as introduced by [16]. Applying (7) to one of the Models (1)-(6) reads as follows: *λ*=1 refers to a model with no response transformation, *λ*=0.5 corresponds to a square-root transformation of the response and *λ*=0 can be interpreted as exponential enlargement of the GA size.

**Model comparison** The main criterion used for our model comparisons was Akaike’s Information Criterion (AIC) [19]. More specifically, our aim was to choose the transformation parameter *λ* that minimized AIC on the analysis data set while assuring that the assumptions of Models (1) to (6) were best possibly met, in particular the normality of the residuals. The AIC is defined by AIC=−2· log(*L*)+2·*n*_params_, where *L* is the likelihood of the model under consideration (evaluated at the maximum likelihood estimate) and *n*_params_ denotes the number of parameters used in the model. As we compared models with a transformed response, we applied the density transformation theorem to compute the likelihood *L*.

**Maximum likelihood estimation** The estimation of the model parameters was performed by maximum likelihood (ML) estimation. ML estimation was carried out for a grid of fixed transformation parameters *λ* using the transformed GA size values. Subsequently, the likelihoods were compared and the transformation parameter referring to the model with minimum AIC was considered best.

We initially assumed that there was an “optimal” value *λ* for which the transformed atrophy size given the random effects followed a normal distribution. In addition, we briefly considered random effects with an unspecified mixing distribution as a non-parametric cross-check. The two approaches will be described in the next paragraphs.

**Normally distributed random effects** As noted above, the linear model in (6) imposes numerous side conditions on the slope parameters associated with *t*, ***x***_*i*_,***x***_*i*_*t*,*γ*_*i*_, and ***x***_*i*_*γ*_*i*_. In order to fit Model (6) using readily available software for the estimation of the slope parameters (without side conditions, such as the R add-on package **lme4**[20], version 1.1-25), we propose to iterate the following steps:

(i) For given estimates $\hat {\beta }$ and $\hat {\boldsymbol {\beta }}_{x}$ compute the values of the working covariable $\tilde {x}_{i} := \hat {\beta } + \hat {\boldsymbol {\beta }}_{x}^{\intercal } \boldsymbol {x}_{i}$.
(ii) Fit the linear mixed-effects model
  8 $$ y_{{ijt}} = \beta t + \boldsymbol{\beta}_{x}^{\intercal} \boldsymbol{x}_{i} t + \mu_{\Delta} \tilde{x}_{i} + \tilde{x}_{i} \gamma_{i} + \tilde{x}_{i} \zeta_{j} + \epsilon_{{ijt}}   $$
  to obtain updates of the coefficient estimates of $\hat {\mu }_{\Delta }, \hat {\beta }$, and $\hat {\boldsymbol {\beta }}_{x}$. Note, that Model (8) is just a re-formulation of Model (6) that can be fitted without side conditions on its slope parameters. For the fitting procedure a fixed intercept term is added to increase computational stability and to relax the condition that the empirical mean of estimated random effects terms is forced to be zero.

The starting values for $\hat {\beta }$ and $\hat {\boldsymbol {\beta }}_{x}$ in Step (i) may be obtained from (8) with an initial value of $\tilde {x}_{i} = 1$. As demonstrated in the supplementary materials (see Additional file 1), repeated execution of (i) and (ii) will typically converge to the final estimates after less than 20 iterations.

**Random effects with unspecified mixing distribution** As an alternative to mixed-effects modeling with normally distributed terms, Almohaimeed et al. [21] proposed to consider a nonparametric maximum likelihood (NPML) approach. This approach approximates the distribution of each random effect by a discrete distribution with finite number of mass points *K*. It then uses an expectation-maximization algorithm to find the nonparametric maximum likelihood estimate. Here, the NPML approach is used to verify the optimal transformation parameter obtained from modeling with normally distributed random effects.

### Age-of-onset estimation

**Model without covariables** As defined by [22], a diagnosis for GA can be given at a minimum lesion diameter of 250 µm and thus a lesion area of 0.05mm^2^. Based on this specification and denoting *λ*_*opt*_ as the value of *λ* that is optimal w.r.t. AIC, the time $\hat {t}_{0_{{ij}}}$ at which the atrophy size was $\hat {y}_{ijt_{0}} = 0.05 [\text {mm}^{2}]$ (i.e. $ \hat {y}^{(\lambda)}_{ijt_{0}} = \lambda _{{opt}}^{-1} \cdot (0.05^{\lambda _{{opt}}}-1) $) can be obtained by solving the model equation of the transformed mixed-effects Model (3) for *t*:

9 $$ \hat{t}_{0_{{ij}}} = \frac{ \lambda_{{opt}}^{-1} \cdot \left(0.05^{\lambda_{{opt}}} -1 \right) - \left(\hat\mu_{\theta} + \hat{\zeta_{j}} + \hat{\alpha}_{i} \right)}{\hat{\beta}},   $$

where $\hat {\beta }$ and $\hat {\mu }_{\theta }$ denote the ML estimates of *β* and *μ*_*θ*_, respectively, and $\hat {\zeta }_{j}$ and $ \hat {\alpha _{i}}$ denote the realizations of the random effect terms. As a consequence, subtracting the estimated time $\hat {t}_{0_{{ij}}}$ from the patient’s age at study entry results in the estimated age-of-onset of GA in the *i*-th eye of patient *j*. Remark: While from a modeling perspective a theoretical atrophy size of $y_{ijt_{0}}={0} \text {mm}^{2}$ could be defined at the time of disease onset, we will focus on the clinically relevant definition $\left (y_{ijt_{0}}={0.05} \text {mm}^{2}\right)$ here. For *y*=0 it holds that $t_{0_{{ij}}} = \Delta _{{ij}} =\frac {1}{\beta } \cdot (\mu _{\theta } + \zeta _{j} + \alpha _{i} $).

**Model with covariables** Analogous to (9) one can estimate the ages of GA onset of the study eyes in a model with additional covariables. From Eq. 8 one obtains

10 $$ \hat{t}_{0_{{ij}}} = \frac{ \lambda_{{opt}}^{-1} \cdot \left(0.05^{\lambda_{{opt}}} -1 \right) - \tilde{x}_{i} \left(\hat\mu_{\Delta} + \hat{\zeta_{j}} + \hat{\alpha}_{i} \right)}{\tilde{x}_{i}}   $$

where $\tilde {x}_{i} := \hat {\beta } + \hat {\boldsymbol {\beta }}_{x}^{\intercal } \boldsymbol {x}_{i}$ contains the parameters obtained from ML estimation.

### Prediction

Evaluating a model and its coefficients only on a transformed scale is challenging as the linearity of the predictor-response relationships in Models (5) and (6) only holds on the transformed scale but not on the original scale of the response (provided that *λ*≠1). As a consequence, the calculation of the expected GA size $\mathbb {E}(y|\boldsymbol {x})$ – and hence any prediction of expected disease progression – cannot be done in an unbiased way by a simple back-transformation.

To see this, consider a non-linear Box-Cox transformation *f*(*y*) with an arbitrary parameter *λ*≠1 and, where existent, the corresponding inverse Box-Cox transformation *f*^−1^(*y*). Further, let *f*(*y*_*ijt*_|***x***_*i*_)=*z*_*ijt*_+*ε*_*ijt*_, where ${z}_{{ijt}} := \mathbb {E}(f(y_{{ijt}}|\boldsymbol {x}_{i}))$ and *ε*_*ijt*_ denote the linear predictor and the residual, respectively in one of the above models. A naive back-transformation would directly take the inverse of the linear predictor, i.e. *f*^−1^(*z*_*ijt*_), which differs from the desired expected GA size value $\mathbb {E}(y_{{ijt}}|\boldsymbol {x}_{i}) = \mathbb {E}\left (f^{-1}\left ({z}_{{ijt}}+ {\epsilon }_{{ijt}}\right)\right)$ by Jensens’s inequality [23]. In other words, $f^{-1}\left (\mathbb {E}\left (f\left (y_{{ijt}}|\boldsymbol {x}_{i}\right)\right)\right) \neq \mathbb {E}(y_{{ijt}}|\boldsymbol {x}_{i})$. To address this issue and to obtain unbiased predictions of the GA size, we propose to sample *r*=10,000 residuals from the empirical distribution $\hat {\epsilon }_{1},..., \hat {\epsilon }_{r}$ in the respective fitted model. The expected atrophy size on the original scale can then be estimated by $\widehat {\mathbb {E}(y_{{ijt}}|\boldsymbol {x}_{i})} := \frac {1}{r}\sum _{u = 1}^{r} f^{-1}\left (\hat {z}_{{ijt}} + \hat {{\epsilon }}_{u} \right)$, where $\hat {z}_{{ijt}}$ denotes the fitted value of *f*(*y*_*ijt*_|***x***_*i*_).

## Results

In this section, we present the results obtained from fitting Models (2), (3) and (6) to the analysis data set (150 eyes of 101 patients). Missing values in the covariables were imputed using the R package **mice** [24] with one imputation run. Fitting was done using **lme4** [20] with the algorithm described above.

### Modeling of GA size trajectories

**Fig. 2:**
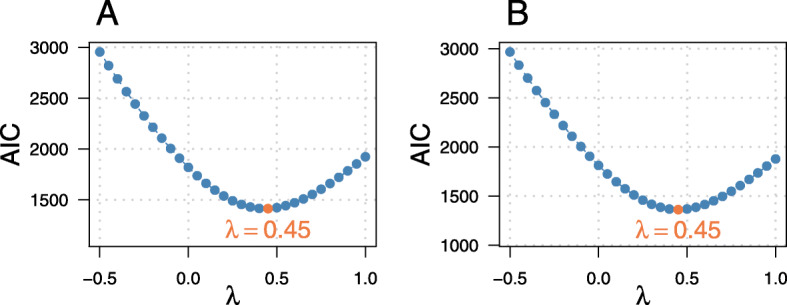
Determination of the optimal Box-Cox transformation. For each value of the transformation parameter *λ*, parametric mixed-effects models **A** without covariables as in Model (3) and **B** with covariables as in Model (6) were fitted to the analysis data set. Model fitting was performed using the R package **lme4**. The orange dot indicates the optimal fit, which was achieved at *λ*_*opt*_=0.45. For Model (3), the optimal AIC value was *AIC*_*λ*=0.45_=1413.69 and for Model (6) the optimal AIC value was *AIC*_*λ*=0.45_=1347.78

**Determination of the transformation parameter** In order to determine the optimal value of the transformation parameter *λ*, we evaluated linear mixed-effects models of the forms (3) and (6) on the analysis data set. Box-Cox-transformed responses with varying values of *λ* were considered in each of the models. As seen in Fig. 2A, the minimum AIC value was reached at *λ*_*opt*_=0.45 in the model without covariables. The model with covariables also yielded an optimal AIC value at *λ*_*opt*_=0.45 (Fig. 2B).

**Fig. 3:**
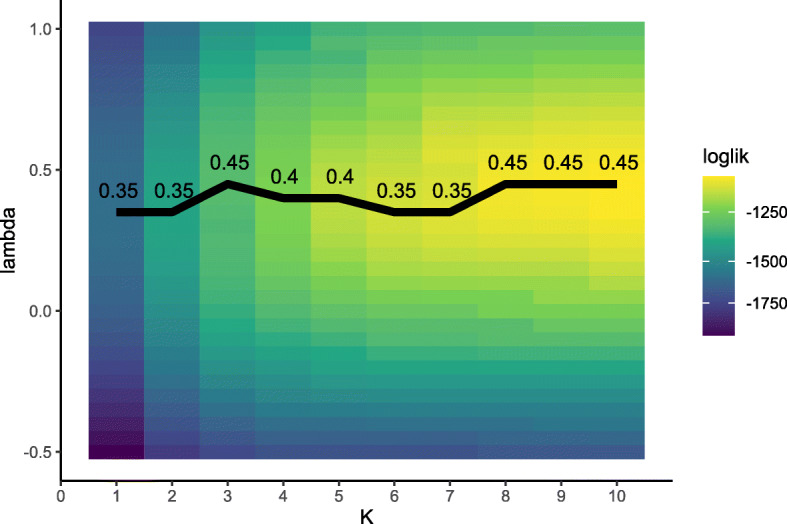
Optimization using NPML approach. Log-likelihood values obtained from fitting Model (2) to the analysis data set with the NPML method, as implemented in the R package **boxcoxmix** [21]. The black line indicates the optimal values of the transformation parameter *λ* for varying numbers of mass points *K*. The corresponding values can be found in Table 2

The NPML approach led to similar results for the optimal value of *λ* in the setting without covariables. As seen in Fig. 3, the obtained values for the optimal *λ* ranged between 0.35 and 0.5. For a larger number of mass points (*K*>7) the same optimal *λ* (= 0.45) as in the parametric approach was found.

**Table 2:** Optimization using NPML approach

K	loglik	*λ*_*opt*_	AIC_*bm*_
1.00	-1593.02	0.35	3192.04
2.00	-1402.21	0.35	2814.41
3.00	-1310.29	0.45	2634.57
4.00	-1230.15	0.40	2478.30
5.00	-1164.85	0.40	2351.70
6.00	-1142.70	0.35	2311.39
7.00	-1127.57	0.35	2285.14
8.00	-1107.63	0.45	2249.26
9.00	-1102.10	0.45	2242.19
10.00	-1096.30	0.45	2234.60

The table presents the optimal values of the transformation parameter *λ* that were obtained from fitting Model (2) with the **boxcoxmix** package [21] using the analysis data set. In addition, the respective log-likelihood and AIC_*bm*_ values (evaluated at the optimal *λ* values) are shown for varying numbers of mass points *K*. Following [21], the information criterion was defined as AIC_*bm*_=−2 log(*L*)+2·(*p*+2*K*). Hence the AIC values in the fourth column cannot be directly compared to the AIC values presented in Fig. 2

**Fig. 4:**
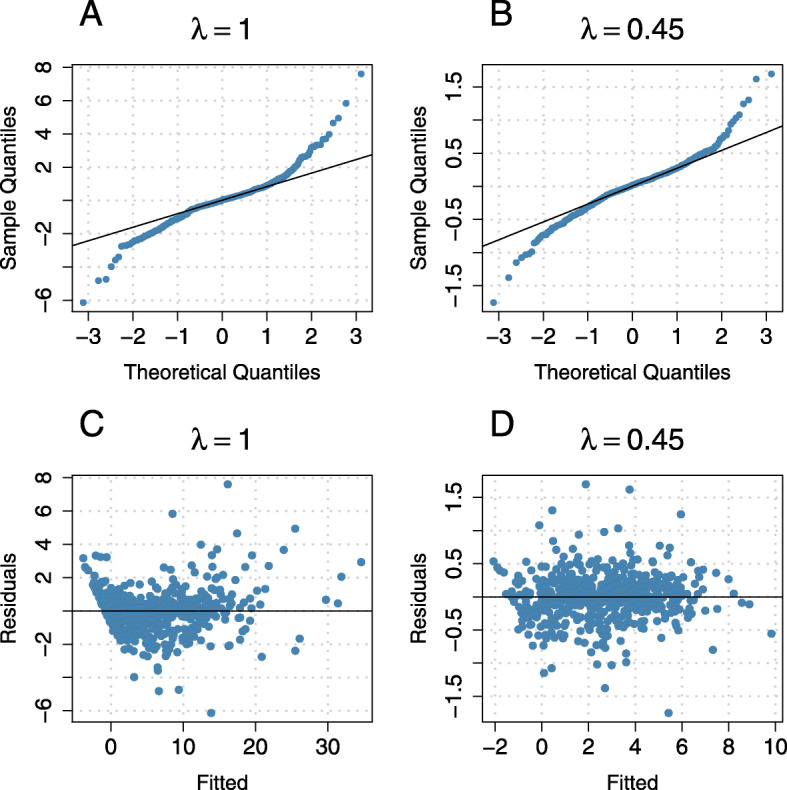
Distribution of residuals. Residual diagnostics for Model (6) with transformation parameter *λ*=1 (left column) and optimal transformation parameter *λ*=0.45 (right column). Note that *λ*=1 corresponds to a model with untransformed response. Panels **A** and **B** present normal quantile-quantile plots of the estimated residuals that were obtained from fitting Model (6) to the analysis data set. Panels **C** and **D** contain plots of estimated residuals vs. fitted values (fitted values include random effect terms)

**Normality of the residuals** Figure 4 shows the residual diagnostics obtained from fitting Model 6 to the analysis data, including hypercholesterolemia and hypertesnsion as risk factors. It is seen that even after transformation the fitted residuals were not normally distributed. However, homoscedasticity was better met after transformation with *λ*_*opt*_=0.45. Furthermore, the distribution of the residuals was less skewed after transformation.

**Fig. 5:**
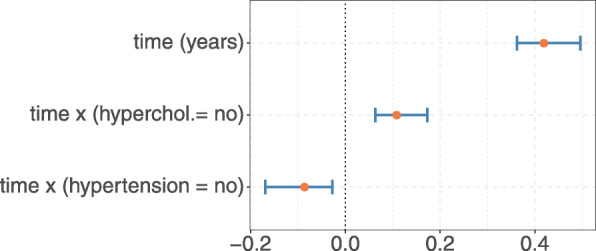
Analysis of risk factors in the analysis data set. The plot shows the coefficient estimates (red dots) that were obtained from fitting Model (6) with transformed response (*λ*=0.45) to an imputed version of the analysis data set. Bootstrap 95% confidence intervals are indicated by blue lines. For further details see Table 3

**Effects of risk factors** As shown in Fig. 4, the residuals obtained from fitting Model 6 to the analysis data set did not perfectly follow a normal distribution, even after transformation of the response. Therefore, inference procedures that rely on asymptotic normality may not be the best choice to investigate the effects of risk factors on (transformed) GA size. To address this issue, we used a bootstrap approach to obtain the 95% confidence intervals of the parameters within Model (6). The results are presented in Table 3 and in Fig. 5. It is seen, that time was associated with the transformed GA size, growing by 0.42 (95% CI [0.36,0.50]) per year. Also the absence of hypercholesterolemia was associated with more rapid enlargement of the lesion size (estimate: 0.11, 95% CI [0.06,0.17]), while a slower progression in patients without hypertension (estimate: −0.09, 95% CI [ −0.17,−0.03]) was found. Note that the estimated coefficients refer to transformed GA size and thus cannot be directly interpreted in terms of an enlargement of the GA size measured in mm^2^.

**Table 3:** Analysis of risk factors in the analysis data set

Variable	Estimate	95% CI	*p*-value
time [in years]	0.42	(0.36, 0.50)	<0.0001
time x (hyperchol.= no)	0.11	(0.06, 0.17)	<0.0001
time x (hypertension = no)	-0.09	(-0.17,-0.03)	0.0004
**Variance Term**	**Estimate**		
Eye:Patient *γ*_*i*_	1.83^2^		
Patient *ζ*_*j*_	4.03^2^		
Residuals *ε*	0.42^2^		

The table presents the coefficient estimates and bootstrap 95% confidence intervals that were obtained from fitting Model (6) with transformed response (*λ*=0.45) to an imputed version of the analysis data set. The model parameter *μ*_*Δ*_, which reflects the mean disease age at study entry, was estimated to be $\hat {\mu }_{\Delta }=4.74$ (95% CI [3.41, 4.83]). *P*-values were obtained using the R package **lmerTest** [25]

Remark: Model fitting was performed on an imputed data set, using the R package **mice** [24] with one imputation. Results obtained from complete case analysis were almost identical.

### Age-of-onset estimation

**Fig. 6:**
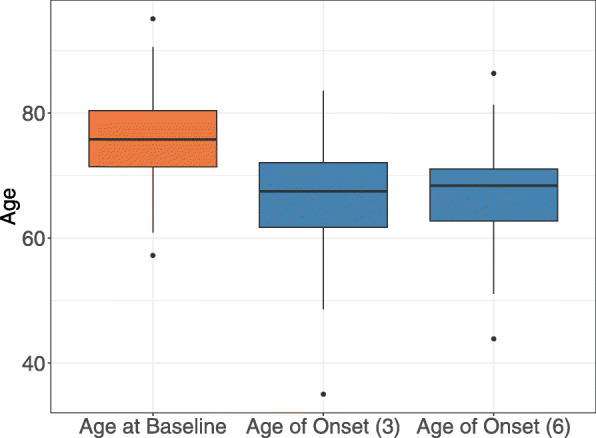
Age-of-onset estimation. Boxplots of the observed ages at baseline (orange) and the modeled ages of disease onset (Models (3) and (6), blue) of the eyes in the analysis data set

Figure 6 presents the estimated ages of disease onset of the study eyes, as obtained from Models (3) (without covariables) and (6) (with covariables). For the simple model without further covariables, the estimated mean age-of-onset was 66.93 (±7.56) years and for the model with covariables the estimated median age-of-onset was 67.21 (±6.49) years. This is in line with previously reported results, e.g. Li et al. [26] estimated the prevalence of GA in people under 64 years to range between 0.1*%* and 0.2*%*, depending on the country.

### Estimation of GA size on the original scale

**Fig. 7:**
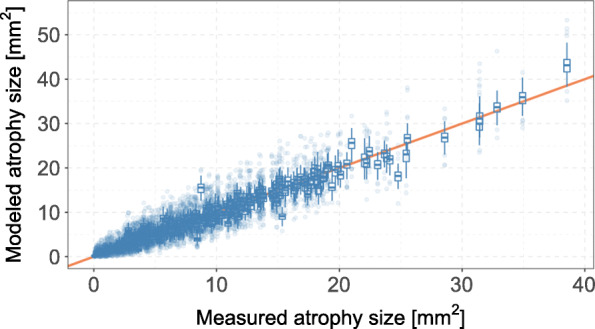
Agreement between the estimated expected GA size values and the measured GA size values modeled distributions show one boxplot per observation and were generated by sampling from the residual distribution of Model (6), followed by a back-transformation of *z*_*ijt*_+*ε*_*u*_ to the original scale. The orange line indicates a perfect fit

To obtain the distribution of GA size on the original scale, we sampled 10,000 times from the empirical distribution of the estimated residuals (obtained from Model (6)) and added these values to the fitted transformed GA size values *f*_*λ*_(*y*) before applying a reverse Box-Cox transformation. The back-transformed expected GA size values are shown in Fig. 7.

**Fig. 8:**
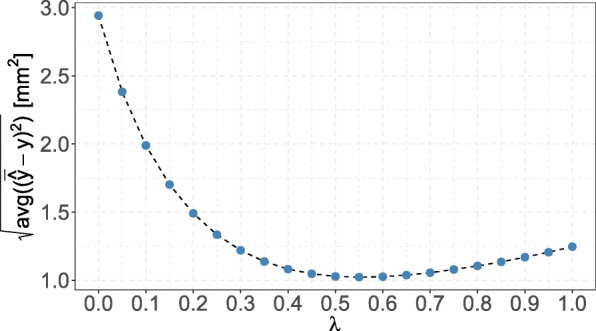
Root mean squared deviation between modeled response and observed values for different transformation parameters. Deviation was measured by $\text {avg}((\bar {\hat {y}} - y)^{2})$. Here, the back-transformed estimate was defined by $$ \hat{y}=\left[ JWFFXGRAPHICS\right]s12874-021-01356-0\mathrm{flba}.\mathrm{eps}{\left[ JWFFXGRAPHICS\right]}^{-1}\left({\hat{y}}^{\left(\lambda \right)}+\hat{\varepsilon}\right) $$ (if [*JWFFXGRAPHICS*]*s*12874−021−01356−0*flbb*.*eps*[*JWFFXGRAPHICS*]^−1^ existed), where [*JWFFXGRAPHICS*]*s*12874−021−01356−0*flbc*.*eps*[*JWFFXGRAPHICS*]^−1^ was the inverse Box-Cox transformation, and $\bar {\hat {y}}$ was its mean (computed from sampling 10,000 times from the fitted residuals). For *λ*=0.45, the deviation was 1.10mm^2^ and a minimal squared deviation was reached at *λ*=0.55 with a deviation of 1.05mm^2^

The root mean squared difference between the observed GA size and the modeled GA size was 1.10mm^2^, implying that estimated expected GA size values deviated by ca. 1mm^2^ on average from the true GA size values. The respective mean squared differences for alternative values of the transformation parameter *λ* are shown in Fig. 8. As can be seen here, the *λ*, that lead to a minimal difference on the original scale, was slightly larger than the optimal *λ*=0.45 obtained by AIC-based methods. However, the variation in the average distances between observed and predicted values was rather small (minimal distance 1.05mm^2^ at *λ*=0.55,1.06mm^2^ at *λ*=0.50, and 1.10mm^2^ at *λ*=0.45).

**Prediction of next observation** In clinical context, a prediction of the next observation of a patient already included in a clinical trial might be of interest. For each observed eye, for which values of more than three visits were present, we predicted the last observation. To this purpose we fitted a model to a training data set excluding the last observation while performance was measured on the last observation. The root mean squared difference between observed atrophy sizes and the mean predicted atrophy sizes was $\sqrt {\text {avg}((\bar {\hat {y}} - y)^{2})} = {1.67} \text {mm}^{2}$.

## Discussion

Despite a high prevalence and extensive research efforts, there are currently no effective standard treatment options for GA. It is therefore essential to develop accurate models for disease progression that enable researchers to efficiently plan and design clinical trials.

In this article, we presented a comprehensive framework for modeling the course of GA size progression in longitudinal observational studies. Our modeling approach was derived from a linear enlargement model using transformed GA size as response variable. As shown in the Results section, the resulting model can be embedded in the class of linear mixed-effects models [27], allowing for the incorporation of risk factors, confounding variables, and measurements taken repeatedly from the same patients and eyes. Since the assumption of linear enlargement imposes numerous restrictions on the model parameters, it is necessary to adapt standard (unrestricted) mixed-effects modeling approaches to the specific structure of the proposed model. To this purpose, we developed an algorithm for GA size modeling that can be implemented using readily available software for fitting linear mixed-effects models.

To obtain the best transformation of GA size, we conducted a systematic search within the class of Box-Cox transformation models that included both parametric and non-parametric approaches. Our experiments yielded an optimal transformation that was close to the square-root function, thereby justifying earlier modeling strategies that assumed linear trajectories of square-root transformed GA size over time [18]. Of note, the square-root transformation has a straightforward interpretation in terms of a linear enlargement of the atrophy radius [15].

A convenient feature of the proposed modeling approach is that it yields estimates of the disease age of the eyes at study entry. This is important because patients can only be included in trials when the disease has already manifested. When applied to the analysis data set consisting of patients included in the FAM-study, disease age at study entry was estimated to range between 3.5 and 13.4 years (Model (6)). These estimates are in line with estimated prevalence values reported in the literature [4], but the resulting ages of disease onset were smaller than previously modeled ages using data partly from the same study [28].

Since the proposed modeling approach employs a transformed response variable, care has to be taken when making predictions of future values of atrophy size. As argued in the Results section, predictions with a naive back-transformation may show a bias due to the non-linearity of the square-root function. To address this issue, we proposed a sampling approach that allows for drawing valid conclusions and making undistorted predictions of GA size on its original scale. In the analysis data set, estimated expected GA size values derived from the proposed model deviated 1.10mm^2^ on average from the respective observed values.

Generally, the model proposed here allows for performing statistical hypothesis tests on a set of risk factors suspected to accelerate or slow down GA size enlargement. This strategy was illustrated in the Results section, where an analysis of a GA patient sample of the FAM study identified significant interaction effects between hypercholesterolemia, hypertension and time. Although a number of studies have shown a link between cardiovascular risk factors and AMD, the role of hypertension, atherosclerosis, high BMI, diabetes mellitus, higher plasma fibrinogen and hyperlipidaemia remain equivocal owing to inconsistent findings (reviewed in [29]). High blood pressure is shown to be associated with lower choroidal blood flow and disturbed vascular homeostasis [30]. Since perfusion deficits in the choriocapillaris, the innermost layer of the choroid, are associated with future GA progression [31], an associate between hypertension and increased GA progression appears biologically plausible. Regarding the association of hypercholesterinemia and decreased GA progression, the biological plausibility remains elusive. The majority of previous studies did not find any relationship between systemic cholesterol levels and progression to early AMD, GA or nAMD (reviewed in [29]), although two studies found an association between serum cholesterol on the development of late stage AMD [32, 33]. Interestingly, one of these studies reported that serum cholesterol levels have a protective effect on the development of nAMD, while they are a risk factor for the development of GA [32]. These observations apparently are in contrast to our results; however, there is evidence that different mechanisms may be involved in driving GA enlargement than those increasing the risk of de novo GA development [6]. Further validation of the risk factors, especially on an external data set, is necessary

While it has been established that so-called nascent GA progresses to manifest GA [34], the trajectory of early GA – prior to the minimum lesion size requirement for clinical trials (e.g., 2.5mm^2^) – is poorly understood. The information derived by this modeling strategy can be used to design future intervention studies, for example regarding the stratification of patient groups and the definition of inclusion criteria. Of note, the proposed modeling approach is not restricted to established epidemiological covariables like hypertension but may also incorporate novel markers of disease progression such as patient-reported outcome measures [35], digital biomarkers, and machine-learning-based scores derived from structural imaging data [36]. The proposed model constitutes a flexible framework to systematically investigate the transition from intermediate to late AMD in large observational studies such as the MACUSTAR study (ClinicalTrials.gov: NCT03349801) [37].

## Conclusions

We have provided a comprehensive framework for modelling the trajectories of uni- or bilateral Ga size progression in longitudinal observational studies. Our analysis shows that a square-root transformation of atropy size is recommended before model fitting. The proposed modelling approach allows for the estimation of age-of-onset, identification of risk factors and prediction of future GA size. The risk factors analyzed here require further validation in an external study population.

## Supplementary Information

**Additional file 1** Supplementary information.

## Data Availability

The datasets generated during and/or analysed during the current study are not publicly available in order to protect the privacy of study participants. However, they are available from the principal investigators of the FAM and DSGA studies on reasonable scientific request.

## Abbreviations

AIC: Akaike’s information criterion
AMD: Age related macular degeneration
avg: Average
BMI: Body max index
CI: Confidence interval
DSGA: Directional spread in geographic atrophy study
FAF: Fundus autoflourescence
FAM: Fundus autoflourescence in AMD study
GA: Geographic atrophy
IQR: Interquartile range
ML: Maximum likelihood
nAMD: Neovascular AMD
NPML: Nonparametic maximum likelihood
RPE: Retinal pigment epithelium
