# Supplementary material for: Modeling of atrophy size trajectories: variable transformation, prediction and age-of-onset estimation
Source: BMC Med Res Methodol. 2021 Aug 17;21:170. doi: 10.1186/s12874-021-01356-0 (PMC8369742; doi:10.1186/s12874-021-01356-0)
Supplement: Supplementary file 1 — Additional file 1 Supplementary information. [file 12874_2021_1356_MOESM1_ESM.pdf]

Modeling of Atrophy Size Trajectories: Variable Transformation, Prediction and Age-of-Onset Estimation

Charlotte Behning[1], Monika Fleckenstein[2], Maximilian Pfau[3], Christine Adrion[4], Lukas Goerdt[5], Moritz Lindner[5], Steffen Schmitz-Valckenberg[2], Frank G Holz[5] and Matthias Schmid[1]

Simulation

Proposed fitting algorithm For model fitting with the R package **lme4** [1], a repeated two-step approach was proposed. We evaluated this approach (described in the methods subsection *Normally distributed random effects*) using a simulation study with 100 runs. The simulation set up was as follows: Each simulation data set contained $N = 200$ eyes. The model contained a time-dependent term $\beta = -0.2$ as well as two continuous uniformly distributed covariables that were are associated with the outcome by $\beta_{x1} = 0.5$ and $\beta_{x2} = -0.3$. Further, the model parameter $\Delta_i := (\mu_\Delta + \gamma_i) \sim \mathcal{N}(\mu_\Delta, \sigma^2_\Delta)$ was included with $\mu_\Delta = 3$ and $\sigma_\Delta = 0.4$. The residuals were simulated with $\sigma_\epsilon = 0.15$.

In each simulation run, the two steps of the proposed algorithm were iterated for 100 rounds.

Figures 1 to 4 show the relationship between the estimated effects and the number of iterations. Convergence was attained after less than 20 iterations. Note that an increase of estimation error was observed in a small number of simulation runs after the 30th iteration. In this scenario the estimates of β_0 and β_{x2} showed an oscillating behaviour. For the estimation of β_{x2}, 4 out of 100 simulation runs showed an oscillation of more than 0.1 after the 30th iteration (18 simulation runs after the 90th iteration).

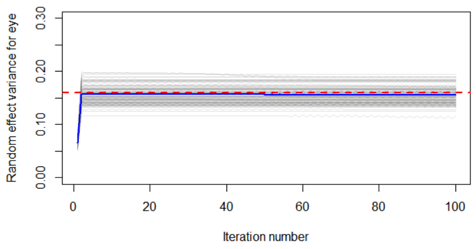

Figure 1 Variance of random effects for 100 different simulation runs. The true random effect variance was $\sigma^2_\Delta = 0.16$, indicated as red line. The mean estimated random effect is indicated by a blue line.

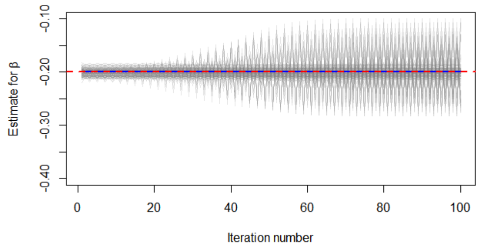

Figure 2 Estimate of β in 100 different simulation runs. The red line indicates the true effect used in the simulation model. The blue line indicates the mean estimated parameter by simulation run.

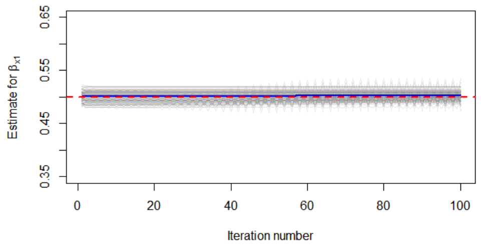

Figure 3 Estimate of β_{x1} in 100 different simulation runs. The red line indicates the true effect used in the simulation model. The blue line indicates the mean estimated parameter.

The **R** version 4.0.3 and **lme4** version 1.1-25 were used in this setup.

Age-of-onset estimation in a simulation setting We evaluated the proposed age-of-onset estimation in a simulation setting. Therefore, a setup with $N = 200$ patients and 4 visits is used. The model parameters were simulated as follows: The model contained a time-dependent term $\beta = 0.5$ as well as one binomially distributed covariable $x1 \sim B(n, 0.5)$ with $\beta_{x1} = 0.2$. Further, t he residuals were simulated with $\sigma_\epsilon = 0.15$ and the random effect parameters

Full list of author information is available at the end of the article

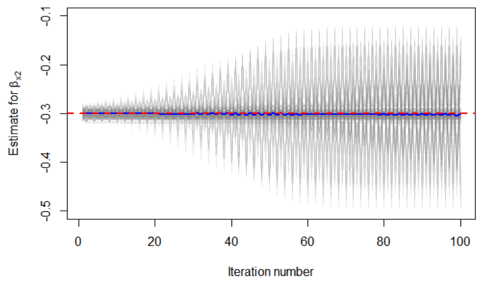

Figure 4 Estimate of β_{x2} in 100 different simulation runs. The red line indicates the true effect used in the simulation model. The blue line indicates the mean estimated parameter by simulation run. For the estimation of β_{x2}, 4 out of 100 simulation runs showed an oscillation of more than 0.1 after the 30th iteration (18 simulation runs after the 90th iteration).

$\Delta_i := (\mu_\Delta + \gamma_i) \sim \mathcal{N}(\mu_\Delta, \sigma_\Delta^2)$ are included with $\sigma_\Delta = 0.4$ and $\mu_\Delta \in \{3, 5, 6, 10\}$.

Enlargement described by the Risk Factors

When a Box-Cox transformation is applied to the response variable, the risk factors have to be interpreted on the scale of the transformed outcome. For example, Figure 6 shows the predicted transformed atrophy size with $\lambda_{opt} = 0.45$ using the fixed effects of the model with the covariates *hypertension* and *hypercholesterolemia* (Model (6) with out random effects). Figure 7, which depicts the estimated expected enlargement described by the risk factors, provides a better understanding of the trajectories on their original scale. Here, a sample of the residuals of Model (6) was added to the fitted GA size values (not including random effects) before back-transformation and subsequent averaging.

Model with $\lambda = 0.5$

Table 1 Model parameters of the analysis data set using $\lambda = 0.5$ The table presents the coefficient estimates and bootstrap 95% confidence intervals that were obtained from fitting Model (6) with transformed outcome ($\lambda = 0.5$) to an imputed version of the analysis data set. Please note, that the model parameter $\mu_\Delta = 4.64$ (95% CI [3.34, 4.72]) rather reflects an estimate for the mean disease age at study entry than a risk factor. P-values are obtained using the package **lmerTest** [2] are not adjusted for multiple testing.

Variable	Estimate	95% CI	p-value
time [in years]	0.45	(0.38, 0.54)	< 0.0001
time x (hyperchol.= no)	0.12	(0.07, 0.20)	< 0.0001
time x (hypertension = no)	-0.09	(-0.18 ,-0.02)	0.0006
**Variance Term**	**Estimate**		
Eye:Patient γ_i	1.79		
Patient ζ_j	4.03		
Residuals ϵ	0.45		

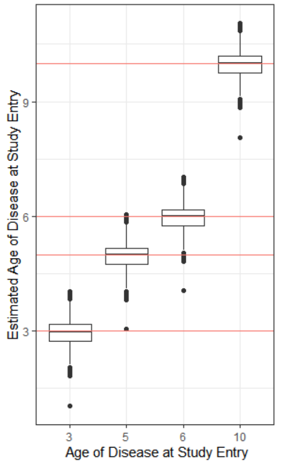

Figure 5 Estimated age of disease at study entry minus $\hat{t}_{0i}$ for different age of disease parameters μ_Δ (shown on the x-axis and indicated as red lines). In the simulation setting, no transformation parameter was used.

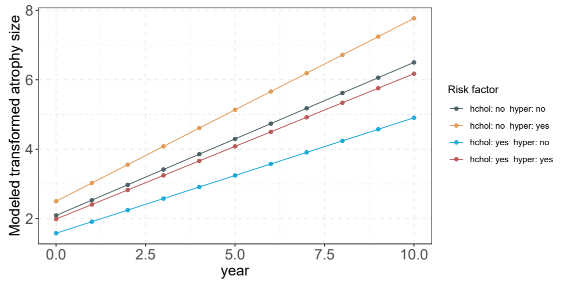

Figure 6 Predicted atrophy sizes on the transformed scale using the fixed effects of the fitted model using the transformation parameter.

As the interpretation of the fitted values is challenging on the transformed scale using a transformation parameter of $\lambda = 0.45$, a transformation using $\lambda = 0.5$ might be better suited in practice. This is because $\lambda = 0.5$ corresponds to a square-root transformation, implying that the model parameters can be interpreted as contributions to the enlargement of a (circular) atrophy radius. The parameter estimates for a model with hypertension and hypercholesterolamia as risk factors are presented in Table 1.

The agreement of the estimated expected GA size values in the model with $\lambda = 0.5$ and the measured atrophy sizes is presented in Figure 8. It is seen that the root mean average distance between the estimated and the observed values was similar to the respective distance computed from the model with $\lambda = 0.45$ (cf. Figure 8 of the publication): For $\lambda = 0.45$, the deviation was $1.10\,\text{mm}^2$, for $\lambda = 0.50$ it was $1.06\,\text{mm}^2$. A

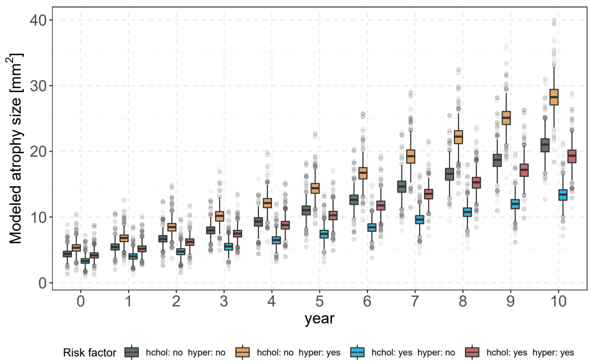

Figure 7 Estimated expected atrophy size on the original (back-transformed) scale using the transformation parameter $\lambda = 0.45$. Random effects were not considered in the back-transformation. Residuals were sampled (1000 times) from the residual distribution of Model (6).

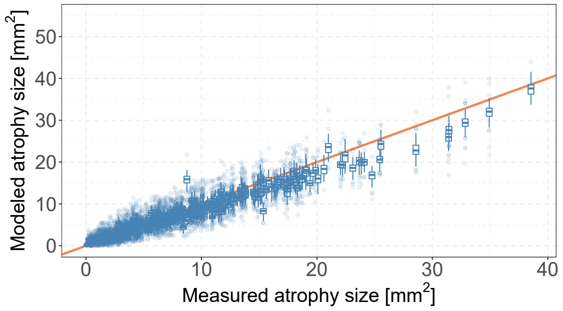

Figure 8 Agreement of the estimated expected GA size values with the measured values Modeled atrophy sizes obtained from fitting Model (6) with covariables to the analysis data set, using a transformation parameter of $\lambda = 0.5$. Predictive distributions show one boxplot per observation and were generated by sampling from the residual distribution of Model (6), followed by a back-transformation of $z_{ijt} + \epsilon_u$ to the original scale. The orange line indicates a perfect fit.

minimal squared deviation was reached at $\lambda = 0.55$, with a deviation of $1.05\,\mathrm{mm}^2$.

Prediction of the last observation Analogous to the main manuscript, we fitted a model with the covariables hypertension and hypercholestorlemia and $\lambda = 0.5$ to a training data set excluding the last observation of each eye. Subsequently, predictions were made for the last observations and compared to the respective true GA size values. The root mean squared difference between observed atrophy sizes and the mean predicted atrophy sizes was $\sqrt{\mathrm{avg}((\bar{\hat{y}} - y)^2)} = 1.59\,\mathrm{mm}^2$.

Age-of-onset estimation For the simple model with $\lambda = 0.5$ without further covariables, the estimated mean age-of-onset was 67.53 (± 7.56) years and for the model with covariables the estimated median age-of-onset was 68.12 (± 6.49) years. These estimates were very close to the respective values obtained from the model with $\lambda = 0.45$, which were 66.93 (± 7.56) years for the simple model and 67.21 (± 6.49) years for the model with covariables.

Author details
[1]Department of Medical Biometry, Informatics and Epidemiology, University Hospital Bonn, Venusberg-Campus 1, 53127 Bonn, Germany. [2]John A. Moran Eye Center, University of Utah, Salt Lake City, USA. [3]Ophthalmic Genetics and Visual Function Branch, National Eye Institute, Bethesda, MD, USA. [4]Institute for Medical Information Processing, Biometry and Epidemiology, Ludwig-Maximilians-University, Munich, Germany. [5]Department of Ophthalmology, University Hospital Bonn, Bonn, Germany. [6]Please add organization, city, country.

References

1. Bates, D., Mächler, M., Bolker, B., Walker, S.: Fitting linear mixed-effects models using lme4. Journal of Statistical Software **67**, 1–48 (2015). doi:10.18637/jss.v067.i01
2. Kuznetsova, A., Brockhoff, P.B., Christensen, R.H.B.: lmerTest package: Tests in linear mixed effects models. Journal of Statistical Software **82**(13), 1–26 (2017). doi:10.18637/jss.v082.i13
